# On the measurement and correlates of plate clearing: examining a German version of the Plate Clearing Tendency Scale

**DOI:** 10.1007/s40519-022-01433-3

**Published:** 2022-06-30

**Authors:** Tina Nill, Adrian Meule

**Affiliations:** 1 Institute of Psychology, University of Würzburg, Würzburg, Germany; 2 Department of Psychiatry and Psychotherapy, University Hospital, LMU Munich, Munich, Germany; 3 Schön Klinik Roseneck, Am Roseneck 6, 83209 Prien am Chiemsee, Germany

**Keywords:** Plate clearing, Body mass index, Food waste, Habit, Dieting, Dieting success

## Abstract

**Purpose:**

Plate clearing—eating a meal in its entirety—is common and may be a factor contributing to obesity. For the assessment of individual differences in plate clearing tendencies, Robinson et al. (Obesity 23:301–304, 2015) developed the Plate Clearing Tendency Scale (PCTS). However, little is known about the psychometric properties of this scale and its correlates.

**Methods:**

In the current study, participants (*N* = 207, 76% female) completed a German translation of the PCTS and other questionnaires online.

**Results:**

A one-factor structure had good model fit and the PCTS had acceptable internal reliability and good test–retest reliability across an average of four and a half weeks. Higher plate clearing tendencies related to more frequent parental encouragement to clear one’s plate in childhood and to stronger food waste concerns but were unrelated to sex, body weight, self-control, and eating behaviors. However, higher plate clearing tendencies related to higher body weight in unsuccessful dieters.

**Conclusion:**

The current study shows that the PCTS has sound psychometric properties and that plate clearing tendencies appear to be largely driven by food waste concerns and not by automatic eating habits or low eating-related self-control. In dieters, however, high plate clearing tendencies may contribute to low dieting success and hinder weight loss.

**Level of evidence:**

No level of evidence, basic science.

## Introduction

Humans tend to eat larger amounts of food with increasing portion sizes (the so-called portion size effect [1]). While different mechanisms have been discussed [2], one factor contributing to the portion size effect may be that humans have a general tendency to eat meals in their entirety, that is, to clear their plate. Plate clearing is very common. For example, 91% of participants indicated that the last reported meal was eaten in its entirety in a study by Fay et al. [3]. Similarly, at least 90% of self-selected food portions were consumed in 86% of meals in a study by Hinton et al. [4]. Although the majority of individuals seems to clear their plate, individual differences in plate clearing tendencies have been found. For example, studies have shown consistently that plate clearing occurs more likely in men than in women [3, 4]. While the mechanisms behind this sex difference are unclear, it has been speculated that men may apply simpler rules of thumb than women by planning to eat the majority of food served at a meal and women’s perceptions of a normal-sized portion tend to be smaller than men’s [5].

### Measuring plate clearing: the Plate Clearing Tendency Scale

Robinson et al. [6] developed a brief self-report measure for the assessment of plate clearing tendencies. While no particular name for this scale has been used to date, this paper will refer to it as the Plate Clearing Tendency Scale (PCTS). The PCTS has five items with higher total scores indicating greater plate clearing tendencies. Internal reliability was good in several studies (*α* > 0.80 [5–9]). Males tend to have higher scores than females and higher scores relate to more concerns over food waste and more frequent parental encouragement to clear one’s plate during childhood [5–8]. While habitual plate clearers as identified by the PCTS showed higher food intake in a laboratory study [9], findings on body weight have been mixed. Specifically, some studies reported small positive associations between PCTS scores and body mass index (BMI, [6, 7]) but PCTS scores and BMI were unrelated in other studies [8, 9].

### Open questions about the PCTS’s psychometric properties and correlates

While the PCTS represents a useful measure for the investigation of plate clearing, there are several open questions about the scale’s psychometric properties and correlates. First, although the scale usually has good internal reliability, its assumed one-factor structure has not been formally tested. Second, although the scale intends to measure plate clearing habits (thus assuming that plate clearing tendencies are stable over time), test–retest reliability has not been examined yet. Third, previous studies have focused on sociodemographic and anthropometric correlates of the scale and concerns over food waste [6–9]. Thus, little is known about other psychological and eating behavior-related correlates of the scale. For example, while one factor contributing to a tendency to clear one’s plate may be concerns over food waste, other factors that are related to, for example, low eating-related self-control have not been considered yet. In the current study, we addressed these open questions regarding the PCTS’s psychometric properties and correlates using a German translation of the scale in a convenience online sample.

### Aims and hypotheses of the current study

#### Psychometric properties of the PCTS

Based on previous studies [5–9], we expected that the scale would show good internal reliability. As the scale was conceptualized as a unidimensional measure, we also expected that a one-factor structure would have good model fit. Because the items ask respondents about their plate clearing tendencies in general, we expected that the scale would have good test–retest reliability across several weeks. Moreover, other self-report measures that supposedly assess eating-related traits have been found to be influenced by current states such as hunger [10]. Thus, as an additional indication for stability of PCTS scores, we expected that they would be unrelated to momentary states such as food deprivation (i.e., time since the last meal) and current food craving and hunger.

#### Correlates of the PCTS

We expected to replicate relationships with variables that have been examined in previous studies (i.e., higher scores in men vs. women and positive correlations with BMI, parental encouragement to clear one’s plate in childhood, and concerns over food waste). In addition, we explored associations with age and several psychological and eating-related constructs: trait food cravings, eating disorder psychopathology, self-control, habit strength, and intuitive eating. As plate clearing tendencies have been conceptualized as a habit [7] and as plate clearers tend to clear their plate independent of portion size [9], we expected that higher PCTS scores would relate to higher eating-related habit strength (capturing automatic aspects of eating) and lower intuitive eating (capturing, e.g., less reliance on physical hunger signals). In addition, self-reported frequency of parental encouragement to clear one’s plate in childhood related to higher eating-related disinhibition scores in adults [11], suggesting that the tendency to clear one’s plate might also be related to low self-control. Thus, we expected that higher PCTS scores would relate to higher trait food craving scores (which are highly correlated with disinhibition scores, [12]) but might also relate to more general eating disorder psychopathology and low self-control.

In one study [9], higher PCTS scores related to higher self-reported dietary restraint as measured by the Dutch Eating Behavior Questionnaire, suggesting that plate clearing tendencies may indeed relate to other eating styles. However, restrained eating scores do not provide any information whether a person is actually restricting food intake (i.e., is currently dieting) or not [13–15] and, if so, whether that person’s dieting attempt is successful or unsuccessful [16]. Thus, we examined the relationship between PCTS scores as a function of both current dieting status and perceived success in weight regulation. Specifically, previous studies have found that considering interactive effects between current dieting and dieting success provide a more fine-grained analysis than using measures of restrained eating [17–19] and may, therefore, lead to more insightful findings about the relationship between dietary restraint and plate clearing tendencies.

## Methods

### Participants and procedure

A convenience sample was recruited through students’ mailing lists at the University of Würzburg, social media (Facebook, Instagram), the website of the German version of Psychology Today magazine (www.psychologie-heute.de/aktuelles/studienteilnahme.html), and Survey Circle (www.surveycircle.com), which is an online community for support in online research. According to the guidelines by the institutional review board of the LMU Munich (the senior author’s primary affiliation), completely anonymized questionnaire studies are exempt from requiring ethics approval. Data were collected in August and September 2020. The study was advertised as a study on eating behavior and personality and potential participants were directed to the online survey at www.soscisurvey.de. Participants were not offered any compensation. The study’s URL received 577 clicks and 234 individuals started the survey. Participants were informed that the data collection was anonymized, that the data would only be used for scientific, non-commercial purposes, and that they agree to participate by starting the survey. Twenty-seven participants cancelled participation before completion of the PCTS. Thus, data of 207 participants who completed the PCTS were available. Descriptive statistics of all study variables are displayed in Tables 1 and 2.

**Table 1:** Descriptive statistics of categorical study variables

*N* = 207	*n*	%
*Sex*		
Female	158	76.3
Male	49	23.7
*Citizenship*		
Germany	193	93.2
Switzerland	3	1.4
Austria	3	1.4
Other	8	3.9
*Education*		
Pupil	2	1.0
Lower school education	1	0.5
Middle school education	17	8.2
Higher school education	78	37.6
Bachelor’s degree	41	19.8
Master’s degree	49	23.7
Completed vocational training	19	9.2
*Occupation*		
Pupil	5	2.4
Student	74	35.7
Employee	88	42.5
Self-employed	6	2.9
Job-seeking	7	3.4
Retired	6	2.9
Other	21	10.1
*Monthly net income*		
Less than 250€	22	10.6
250–499€	21	10.1
500–999€	33	15.9
1000–1499€	31	15.0
1500–1999€	29	14.0
2000–2999€	34	16.4
3000–3999€	10	4.8
4000–4999€	6	2.9
5000€ or more	3	1.4
Don’t want to answer	18	8.7
*Type of diet*		
Vegan	13	6.3
Vegetarian	43	20.8
Pescetarian	15	7.2
Omnivorous	136	65.7
*Dieting status*		
Yes	84	40.6
No	123	59.4

**Table 2:** Descriptive statistics of continuous study variables and correlations with the Plate Clearing Tendency Scale

Variable	*n*	*M*	SD	Range	*ω*	*r*	*p*
Plate Clearing Tendency Scale (sum scores)	207	19.7	3.87	5–25	.76	–	–
Age (years)	207	29.6	10.3	13–68^a^	–	− .07	.307
Body mass index (kg/m^2^)	207	23.9	5.40	12.9–44.3	–	.05	.507
Parental encouragement to clear one’s plate in childhood	206	68.8	29.0	1–100	–	.21	.003
Food waste concerns	207	87.7	19.5	6–100	–	.50	< .001
Food deprivation (hours)	207	3.99	5.88	0–52.5	–	.003	.964
Food Cravings Questionnaire–State							
Craving subscale (sum scores)	185	24.4	9.73	12–53	.93	.06	.420
Hunger subscale (sum scores)	185	6.02	3.15	3–15	.91	.07	.352
Food Cravings Questionnaire–Trait–reduced (sum scores)	193	40.5	14.2	15–81	.94	.07	.312
Brief Self-Control Scale (mean scores)	182	3.13	0.58	1.6–4.7	.79	− .03	.680
Intuitive Eating Scale–2 (mean scores)	172	3.42	0.64	1.7–4.9	.90	− .01	.894
Perceived Self-Regulatory Success in Dieting Scale (sum scores)	171	12.1	3.81	3–21	.69	− .004	.956
Eating Disorder Examination–Questionnaire–8 (mean scores)	171	2.17	1.58	0–5.9	.91	.09	.232
Self-Report Behavioral Automaticity Index (mean scores)	171	2.64	1.01	1.0–5.0	.86	.07	.333

^a^Note that only one participant indicated to be underaged. All other participants indicated to be at least 18 years old

At the end of the survey, participants who were interested to take part in a follow-up survey were asked to enter an individualized code and their email address, which was stored separately from the other data. This information was provided by 141 participants. They were contacted in October 2020 and asked to complete the follow up survey, which consisted of entering the individualized codes and completing the PCTS. Seventy-four individuals completed the follow-up survey. However, four of the individualized codes could not be matched to the ones provided in the main study and, thus, data of 70 participants were available for analyzing test–retest reliability.

### Measures

#### Sociodemographic, anthropometric, and other information

Participants were asked to report their biological sex, age (in years), citizenship, education, occupation, monthly net income, body height (in cm), body weight (in kg), type of diet, and the time since their last meal (in hours). Note that citizenship, education, occupation, monthly net income, and type of diet were only collected for sample description (Table 1) and not used in further analyses. Current dieting status was assessed with the question “Are you currently restricting your food intake to control your weight (e.g., by eating less or avoiding certain foods)?” [17]. Parental encouragement of plate clearing and concerns about food waste were measured with the items “My parents used to always encourage me to clear my plate when eating” and “I don’t like to see food going to waste” [7]. Responses to these two items were recorded on a rating slider anchored 1 = *strongly disagree* and 100 = *strongly agree*.

#### Plate Clearing Tendency Scale (PCTS)

The PCTS [6] consists of five items: “I always tend to clear my plate when eating,” “I normally finish eating when my plate is empty,” “Before I start eating, I normally plan to finish the serving I am about to eat,” “I rarely leave food on my plate,” and “It is normal for me to have very little food left or an empty plate at the end of a meal.” Responses are recorded on a five-point scale ranging from 1 = *strongly disagree* to 5 = *strongly agree*. Thus, higher total scores represent stronger plate clearing tendencies. We translated the scale into German and a bilingual speaker translated the scale back to English. Discrepancies between the back translation and the original form were discussed and items were adjusted accordingly.

#### Food Cravings Questionnaire–Trait–reduced (FCQ–T–r)

The FCQ–T–r [20] is a 15-item short version of the 39-item FCQ–T [21, 22]. Responses are recorded on a six-point scale ranging from 1 = *never* to 6 = *always*. Thus, higher total scores represent more frequent and intense food cravings.

#### Food Cravings Questionnaire–State (FCQ–S)

The FCQ–S [21, 22] consists of 15 items, 12 of which ask about the intensity of current food craving and three ask about the intensity of current hunger. Thus, we examined these two subscales separately instead of analyzing a total score [23]. Responses are recorded on a five-point scale ranging from 1 = *strongly disagree* to 5 = *strongly agree*. Thus, higher subscale scores represent more intense current food craving and hunger, respectively.

#### Brief Self-Control Scale (BSCS)

The BSCS [24, 25] consists of 13 Items. Responses are recorded on a five-point scale ranging from 1 = *not at all like me* to 5 = *very much like me*. After recoding inversely scored items, higher total scores represent higher self-control.

#### Intuitive Eating Scale–2 (IES–2)

The IES–2 [26, 27] consists of 23 items. Responses are recorded on a five-point scale ranging from 1 = *strongly disagree* to 5 = *strongly agree*. After recoding inversely scores items, higher total scores represent higher intuitive eating.

#### Eating Disorder Examination–Questionnaire–8 (EDE–Q–8)

The EDE–Q–8 [28] is an eight-item short version of the 28-item EDE–Q [29, 30]. Responses are recorded on a seven-point scale ranging from 0 = *no days*/*none of the times*/*not at all* to 6 = *every day*/*every time*/*markedly*. Thus, higher total scores represent higher eating disorder psychopathology.

#### Self-Report Behavioral Automaticity Index (SRBAI)

The SRBAI [31] is a four-item short version of the 12-item Self-Report Habit Index [32]. In previous studies, different response scales have been used, for example, five- versus seven-point scales [33]. We decided to use a five-point response scale anchored 1 = *disagree* and 5 = *agree*. The scale requires specifying the type of behavior that the items should refer to, which we specified as *eating* (“Eating is something… “I do automatically,” “I do without having to consciously remember,” “I do without thinking,” “I start doing before I realize I’m doing it”). Thus, higher total scores represent stronger automatic eating habits.

#### Perceived Self-Regulatory Success in Dieting Scale (PSRS)

The PSRS [34, 35] consists of three items (“How successful are you in watching your weight?”, “How successful are you in losing extra weight?”, “How difficult do you find it to stay in shape?”). Responses are recorded on a seven-point scale anchored 1 = *not successful*/*not difficult* and 7 = *very successful*/*very difficult*. After recoding one inversely scored item, higher total scores represent higher perceived success in weight regulation.

### Data analyses

#### Reliability and factor structure

Internal reliability of the PCTS was evaluated with McDonald’s *ω* as it should be preferred over Cronbach’s *α* [36–39] and was calculated with JASP version 0.13.0 (https://jasp-stats.org; [40]). The one-factor structure of the PCTS was tested with confirmatory factor analysis with JASP version 0.13.0, which uses the R-package *lavaan* (http://lavaan.ugent.be). Diagonally Weighted Least Squares was chosen as estimation method because of the ordinal scale structure [41]. Model fit was considered as good according to the recommendations by Schermelleh-Engel and colleagues [42]: *χ*^2^ ≤ 2*df*, *χ*^2^ *p* value > 0.05, *χ*^2^/*df* ≤ 2, Root Mean Square Error of Approximation (RMSEA) ≤ 0.05, RMSEA *p* value > 0.10, RMSEA left boundary of CI = 0.00, Standardized Root Mean Square Residual (SRMR) ≤ 0.05, Bentler–Bonett Normed Fit Index (NFI) ≥ 0.95, Bentler–Bonett Non-normed Fit Index (NNFI) ≥ 0.97, Comparative Fit Index (CFI) ≥ 0.97, Goodness of Fit Index (GFI) ≥ 0.95. In addition to Pearson’s correlation coefficient, test–retest reliability was evaluated with concordance correlation coefficient and Bland–Altman analysis [43] using the packages *blandr* and *seolmatrix* in *jamovi* version 2.3.0 (https://www.jamovi.org). We also tested whether the length of the time interval between the two measurements influenced test–retest reliability by running a moderated regression analysis with PROCESS version 3.4 for SPSS [44] using PCTS scores at the first measurement as independent variable, PCTS scores at the second measurement as dependent variable, and time interval between measurements (in weeks) as moderator variable.

#### Correlates of plate clearing tendencies

Sex differences in PCTS scores were tested with an independent samples *t*-test. Associations between continuous study variables and PCTS scores were tested with Pearson’s correlation coefficients. Interactive effects of dieting status and PSRS scores on PCTS scores were tested with PROCESS version 3.4 for SPSS [44]. Specifically, a linear regression model was estimated with dieting status, PSRS scores, and their product dieting status × PSRS scores as independent variables and PCTS scores as dependent variable. We also explored whether the interactive effect of dieting status and PSRS scores would not only relate to PCTS scores, but also moderate the relationship between PCTS scores and BMI. For this, a linear regression model was estimated with dieting status, PSRS scores, and PCTS scores as well as all two-way and the three-way interaction as independent variables and BMI as dependent variable. Significant interaction effects were followed up by examining simple slopes at high (84th percentile) and low (16th percentile) PSRS scores. Note, however, that participants were not categorized into groups based on PSRS scores—this pick-a-point approach only serves the purpose of probing the interaction effect [44]. Effects with *p* < 0.05 were considered as significant. The data set of this study is available at https://osf.io/278t5.

## Results

### Reliability and factor structure

**Fig. 1:**
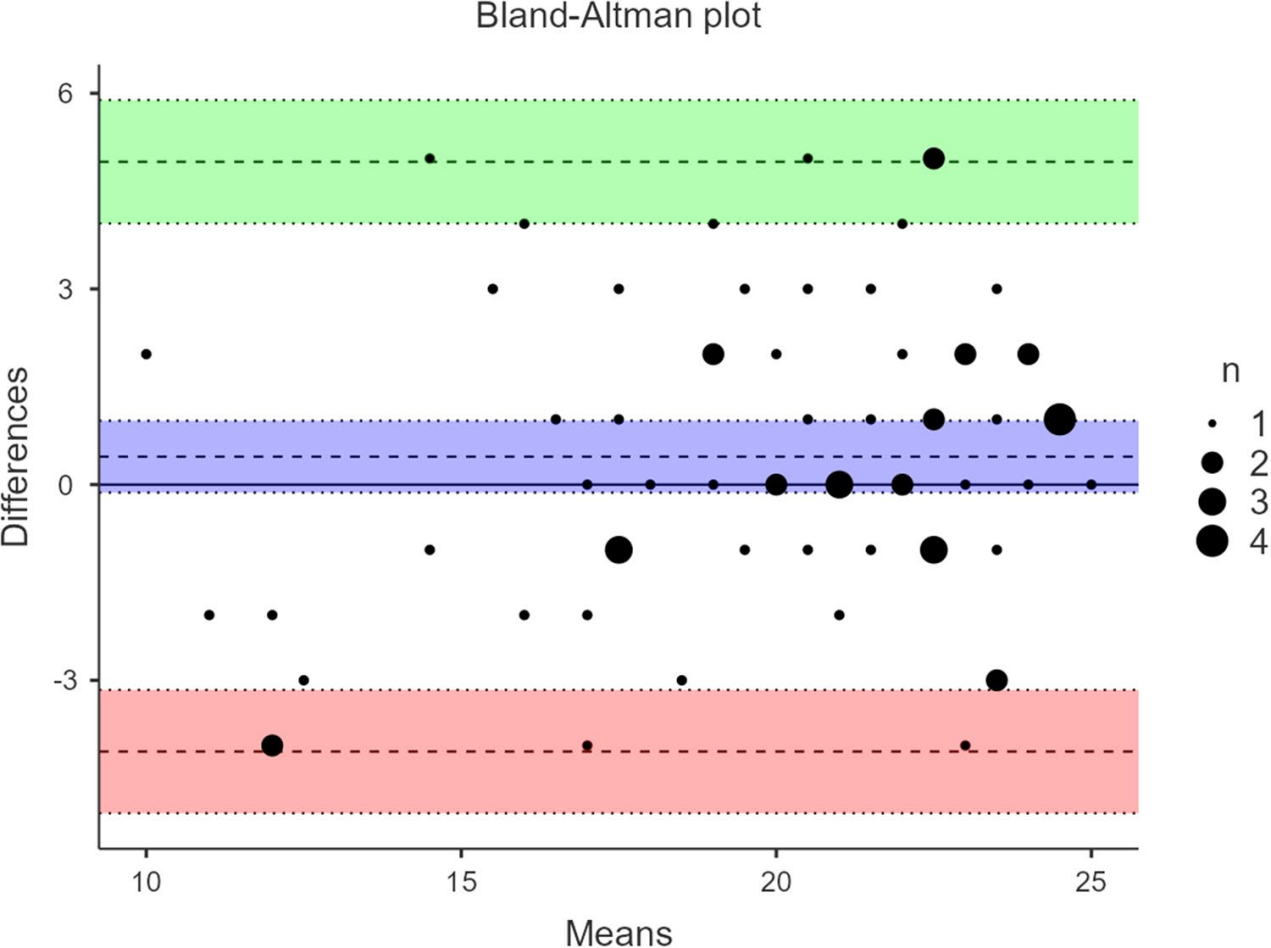
Bland–Altman plot depicting the level of agreement between the first and second measurement of the Plate Clearing Tendency Scale. Averaged scores are plotted on the x-axis and difference scores are plotted on the y-axis. The dashed line in the purple-shaded area (95% CI) represents mean bias. The dashed lines in the green- and pink-shaded areas (95% CI) represent the upper and lower limits of agreement. Overlapping data points are highlighted by larger dot sizes

Internal reliability of the PCTS was acceptable (Table 2) and the one-factor model fitted the data well (*χ*^2^ = 6.14, *χ*^2^ *p* value = 0.29, *χ*^2^/*df* = 1.23, RMSEA = 0.03, RMSEA *p* value = 0.56, RMSEA left boundary of CI = 0.00, SRMR = 0.05, NFI = 0.97, NNFI = 0.99, CFI > 0.99, GFI = 0.99). Internal reliability of the PCTS at follow-up was *ω* = 0.78. Mean time between the two measurements was 4.46 weeks (*SD* = 1.76, Range 1.3–8.3) and test–retest reliability of the PCTS was *r* = 0.83 (95% CI [0.74;0.89]) with a similarly high concordance correlation coefficient of 0.82 (95% CI [0.73;0.88]). The Bland–Altman analysis indicated that mean bias (0.43) was not significantly different from zero (95% CI [− 0.12; 0.98]) and that almost all data points were located within the upper (4.95, 95% CI [4.01;5.89]) and lower (− 4.09, 95% CI [− 5.04; − 3.15]) limit of agreement (Fig. 1). Time between the two measurements did not moderate the association between PCTS scores (*b* = 0.02, *SE* = 0.04, *p* = 0.604).

### Correlates of plate clearing tendencies

Male and female participants did not differ in PCTS scores (*t*_(205)_ = 0.35, *p* = 0.727, *d* = 0.06). Higher PCTS correlated with more frequent parental encouragement to clean one’s plate and with stronger food waste concerns (Table 2). No other study variable was correlated with PCTS scores (Table 2).

**Fig. 2:**
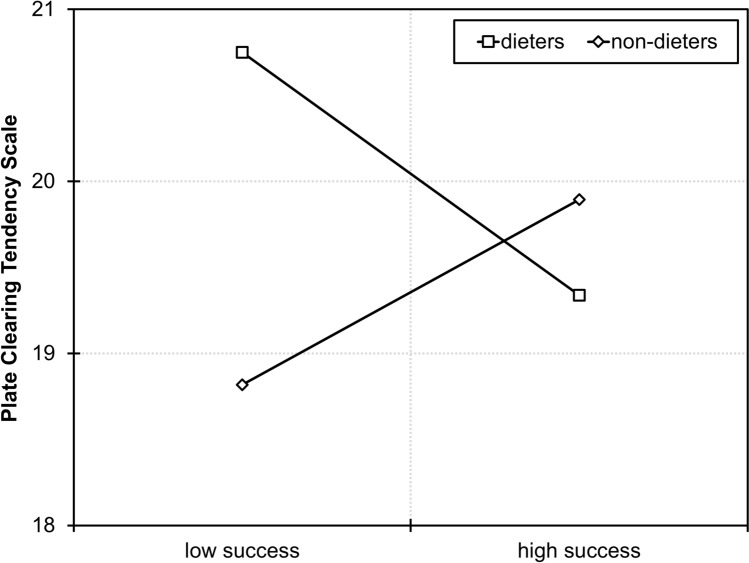
Simple slopes probing the interaction effect of dieting status and scores on the Perceived Self-Regulatory Success in Dieting Scale (PSRS) on scores on the Plate Clearing Tendency Scale. Low and high success represent PSRS scores of 9 (16th percentile) and 16 (84th percentile). Dieters with low PSRS scores had higher plate clearing tendencies than non-dieters with low PSRS scores while there was no difference between groups in those with high PSRS scores. Note, however, that participants were not categorized into groups based on PSRS scores—this pick-a-point approach only serves the purpose of visualizing the interaction effect

The interaction effect of dieting status and PSRS scores on PCTS scores was significant (*b* = 0.36, *SE* = 0.16, *p* = 0.028). Dieters with low PSRS scores had higher plate clearing tendencies than non-dieters with low PSRS scores (*b* =  − 1.93, *SE* = 0.77, *p* = 0.013) while there was no difference between groups in those with high PSRS scores (*b* = 0.56, *SE* = 0.89, *p* = 0.532; Fig. 2).

**Fig. 3:**
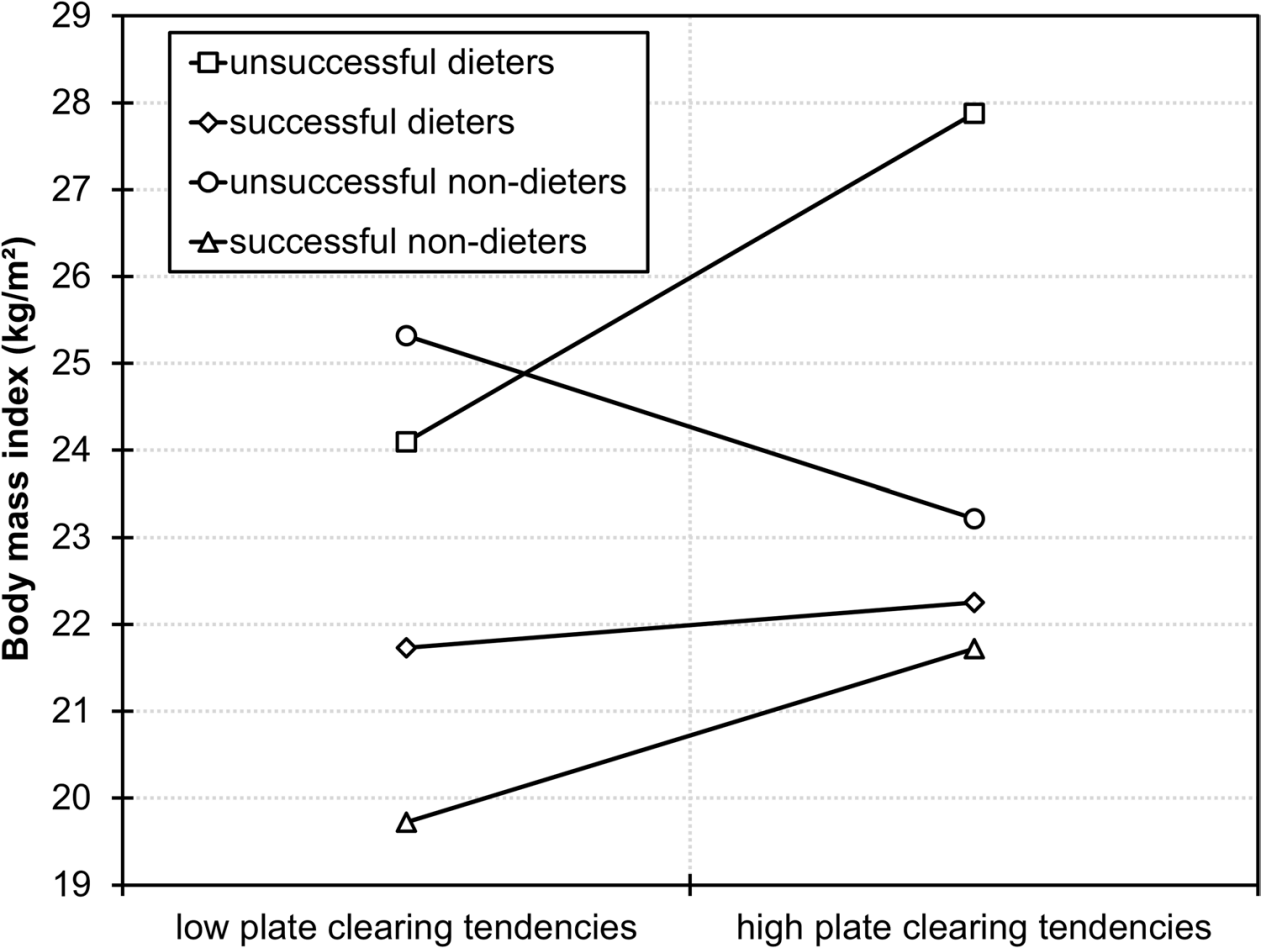
Simple slopes probing the interaction effect of dieting status, scores on the Perceived Self-Regulatory Success in Dieting Scale (PSRS), and scores on the Plate Clearing Tendency Scale (PCTS) on body mass index. Unsuccessful and successful represent PSRS scores of 9 (16th percentile) and 16 (84th percentile). Low and high plate clearing tendencies represent PCTS scores of 16 (16th percentile) and 24 (84th percentile). In unsuccessful dieters, higher plate clearing tendencies related to higher BMI while there was no relationship between PCTS scores and BMI in the other three groups. Note, however, that participants were not categorized into groups based on PSRS and PCTS scores—this pick-a-point approach only serves the purpose of visualizing the interaction effect

The three-way interaction effect of dieting status, PSRS scores, and PCTS scores on BMI was significant (*b* = 0.13, *SE* = 0.05, *p* = 0.008). In dieters with low PSRS scores, higher plate clearing tendencies related to higher BMI (*b* = 0.47, *SE* = 0.20, *p* = 0.020) while there was no relationship between PCTS scores and BMI in dieters with high PSRS scores and in non-dieters with high or low PSRS scores (all *p*s > 0.062; Fig. 3).

## Discussion

### Psychometric properties of the PCTS

The current study is the first to formally test the PCTS’s assumed one-factor structure and its test–retest reliability. While internal reliability of the German PCTS was acceptable and slightly lower than in previous studies that used the English version [5–9], the one-factor structure had good model fit, indicating that the PCTS measures plate clearing tendencies as a unidimensional construct. The scale also had good test–retest reliability across an average of four and a half weeks, suggesting that plate clearing tendencies are relatively stable over time. Finally, stability of PCTS scores was further supported by absent relationships with food deprivation, current food craving, and hunger. Thus, PCTS scores seem to be unaffected by eating-related momentary states.

### Correlates of plate clearing tendencies

In line with previous studies [7, 8], higher plate clearing tendencies related to more frequent parental encouragement to clear one’s plate in childhood and stronger food waste concerns. In contrast to previous studies [5–7], however, plate clearing tendencies were unrelated to sex and BMI in the current study. The absence of sex differences is puzzling as this effect has been consistently reported in the literature, not only in studies that used the PCTS but also in studies that examined plate clearing with other methods [3, 4]. Regarding the relationship between plate clearing and BMI, however, the magnitude of this relationship was small in previous studies [6, 7] and plate clearing did not relate to BMI in other studies that used the PCTS [8, 9] or other methods [3]. Thus—in contrast to previous suggestions [6, 7]—our findings indicate that plate clearing tendencies do not represent an important risk factor for obesity.

Contrary to hypotheses, plate clearing did also not relate to any other eating-related construct (trait food cravings, eating disorder psychopathology, habit strength, and intuitive eating) or general self-control in the current study. Thus, the current findings suggest that plate clearing does not occur as a result of an automatic habit or poor self-control. While this is unexpected, it is also in line with findings indicating that most plate clearing is planned [3].

Although plate clearing tendencies were largely unrelated to other eating behaviors, a relationship with dietary restraint was found, in line with a previous study [9]. Specifically, unsuccessful dieters had the highest PCTS scores and in this subgroup of participants, higher PCTS scores related to higher BMI. Although data were cross-sectional, it might be speculated that plate clearing tendencies—which appear to be established in childhood and remain stable over time—represent an obstacle for dieters to successfully lose weight because they are a steady behavioral pattern that is difficult to change. In addition, these findings highlight the importance of identifying moderators of the relationship between plate clearing tendencies and BMI. That is, the relationship between plate clearing and body weight appears to be a small one at most and, instead, may primarily be found in subgroups of individuals.

### Limitations

Interpretation of the current findings is limited by the use of a convenience sample, self-report measures, and cross-sectional data collection. That is, the current sample was not nationally representative and results might have been influenced by self-selection bias. For example, female and highly educated young adults were overrepresented in the current sample and, thus, findings may not translate to more nationally representative samples. Self-reports may also be biased, for example, self-reported height tends to be overestimated while self-reported weight tends to be underestimated [45]. However, while this bias exists, it has also been found that self-reported height and weight is usually sufficiently accurate in online studies [46, 47]. Furthermore, validity of the PCTS has been supported by associations with laboratory food intake [9] and similar evidence exists for other self-report questionnaires that have been used in the current study [48]. Finally, as data collection was cross-sectional, interpretation of causal relationships need to be considered carefully.

### Conclusion

The current study shows that the PCTS has sound psychometric properties and replicates that plate clearing tendencies relate to parental encouragement to clear one’s plate in childhood. In addition, findings suggest that plate clearing tendencies appear to be largely driven by food waste concerns and not by automatic eating habits or low eating-related self-control. In dieters, however, high plate clearing tendencies may contribute to low dieting success and hinder weight loss.

## What is already known on this subject?

Previous studies have used a brief questionnaire—the Plate Clearing Tendency Scale—for measuring individual differences in plate clearing tendencies. Higher scores were weakly, positively correlated with body mass index and strongly, positively correlated with food waste concerns and parental encouragement to clear one’s plate in childhood.

## What this study adds

This study examines a German version of the questionnaire and shows that it has a unidimensional structure and good test–retest reliability. It also replicates that higher plate clearing tendencies are associated with food waste concerns and parental encouragement to clear one’s plate and adds that they are unrelated to body mass index, other eating behaviors, and self-control. In dieters, however, higher plate clearing tendencies are associated with lower dieting success and higher body weight.

## Declarations

### Data availability

The data set of this study is available at https://osf.io/278t5.
